# Supplementary material for: Biogenic manganese oxide nanoparticle formation by a multimeric multicopper oxidase Mnx
Source: Nat Commun. 2017 Sep 29;8:746. doi: 10.1038/s41467-017-00896-8 (PMC5622069; doi:10.1038/s41467-017-00896-8)
Supplement: Supplementary file 1 — Supplementary Information [file 41467_2017_896_MOESM1_ESM.pdf]

Supplementary Discussion

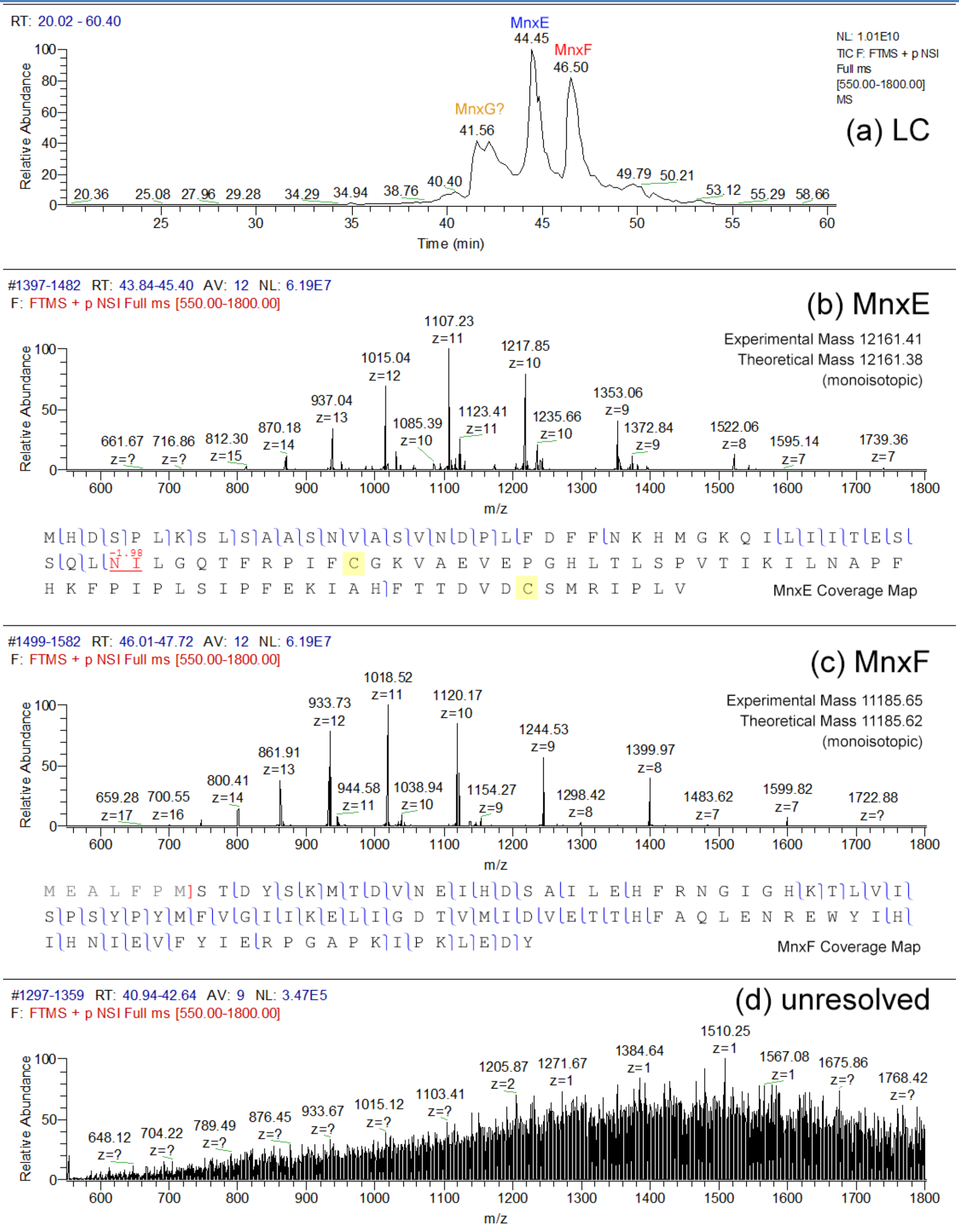

Supplementary Figure 1. Top-down LC-MS data of Mnx.

(a) the total ion current chromatogram, (b) the summed MS spectrum for MnxE, (c) MnxF with the coverage map identified from HCD fragmentation, and (d) the MS spectrum for the unresolved species eluting around 41 min, presumably MnxG which is not fully denatured and had difficultly generating resolved MS peaks in the mass range of detection. The mass shift of –2 Da (highlighted in red with underline) on the intact protein and the region with poor sequence coverage (after residue "SQL" to C-terminus) for MnxE suggest there is a disulfide linkage between the two cysteines (highlighted in yellow). For MnxF, the major species detected had the first 7 residues truncated from the predicted sequence (residues in gray color left to the red bracket). Additional details are in Supplementary Method.

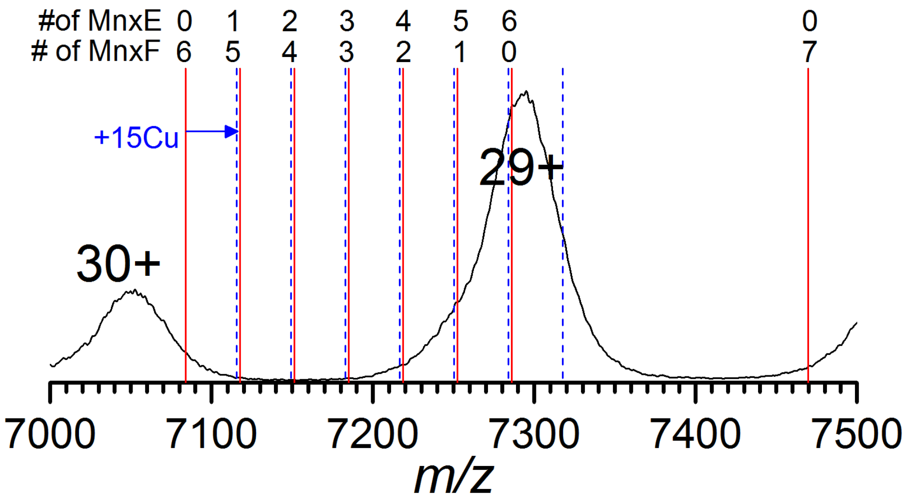

# of MnxE	0	1	2	3	4	5	6	0
# of MnxF	6	5	4	3	2	1	0	7
# of MnxG	1	1	1	1	1	1	1	1
Mass (Da)	205401	206378	207354	208331	209307	210284	211261	216594

Supplementary Figure 2. Experimental mass spectrum of intact Mnx complex (black trace, zoom-in view of the data in Figure 1) and theoretical average masses of the Mnx complex components (lower panel).

The red vertical lines, with corresponding subunit numbers and total mass, also shown in the insert table, represent the Mnx complex with different compositions of MnxE and MnxF subunits overlaid on the native mass spectrum of Mnx complex. Theoretical masses were based on the sequences in Supplementary Note 1 (experimentally verified MnxE and MnxF sequences and the predicted sequence for MnxG). The figure shows that the peak widths of the observed charge states for the native complex (i.e. the charge state of +29 near m/z 7300) could roughly cover the theoretical masses for ~3–4 different combinations of MnxE and MnxF. Assuming each Mnx complex binds 15 Cu, the increased mass of the intact complex (blue dashed vertical lines) will be nearly indistinguishable from apo complex with one more MnxE (and one less MnxF). Additionally, the theoretical mass does not include nonspecifically attached solvent/salt or other modifications which could generate extra uncertainties in determining the composition of the complex. We show in Supplementary Table 2 that MnxG carries a significant amount of extra mass (1 kDa) from the predicted sequence, which could originate from the binding of unknown ligand(s). Therefore, the heterogeneity and limited resolution does not allow direct determination of subunit stoichiometry and the metal binding ratios solely from the measured mass of the intact native Mnx complex.

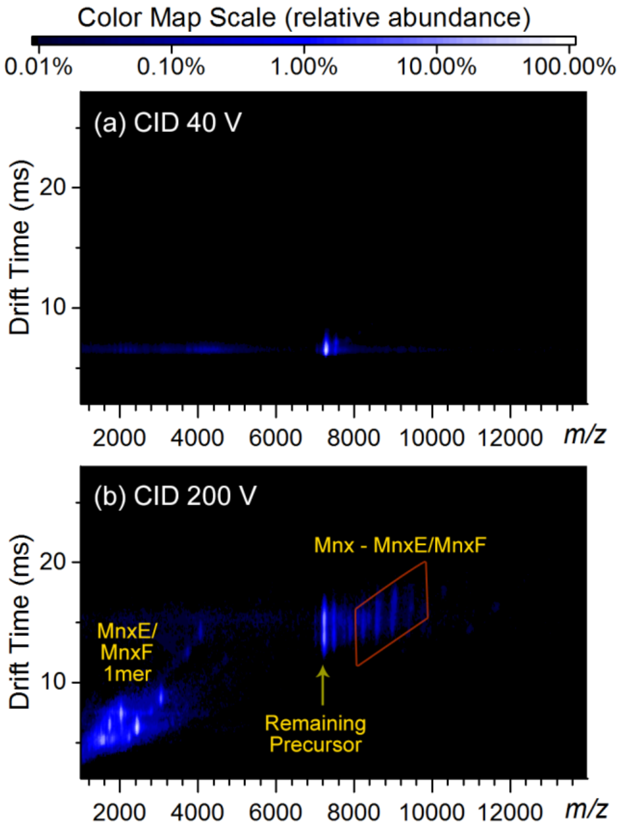

Supplementary Figure 3. CID of isolated Mnx complex.

The 29+ ion at (a) low collision voltage of 40 V and (b) high collision voltage of 200 V, which is the maximum voltage that can be set with standard instrument configuration. In contrast to 40 V SID (Figure 3c) where the MnxEF hexamer is detached from MnxG, no significant dissociation can be seen at 40 V CID. With increasing CID collision voltage, the complex started to unfold with increasing drift times as the monomers started to emerge (data not shown). At 200 V CID, the overall dissociation pathway is still the loss of MnxE/MnxF monomers, which was also observed in CID at 120 V (Figure 3).

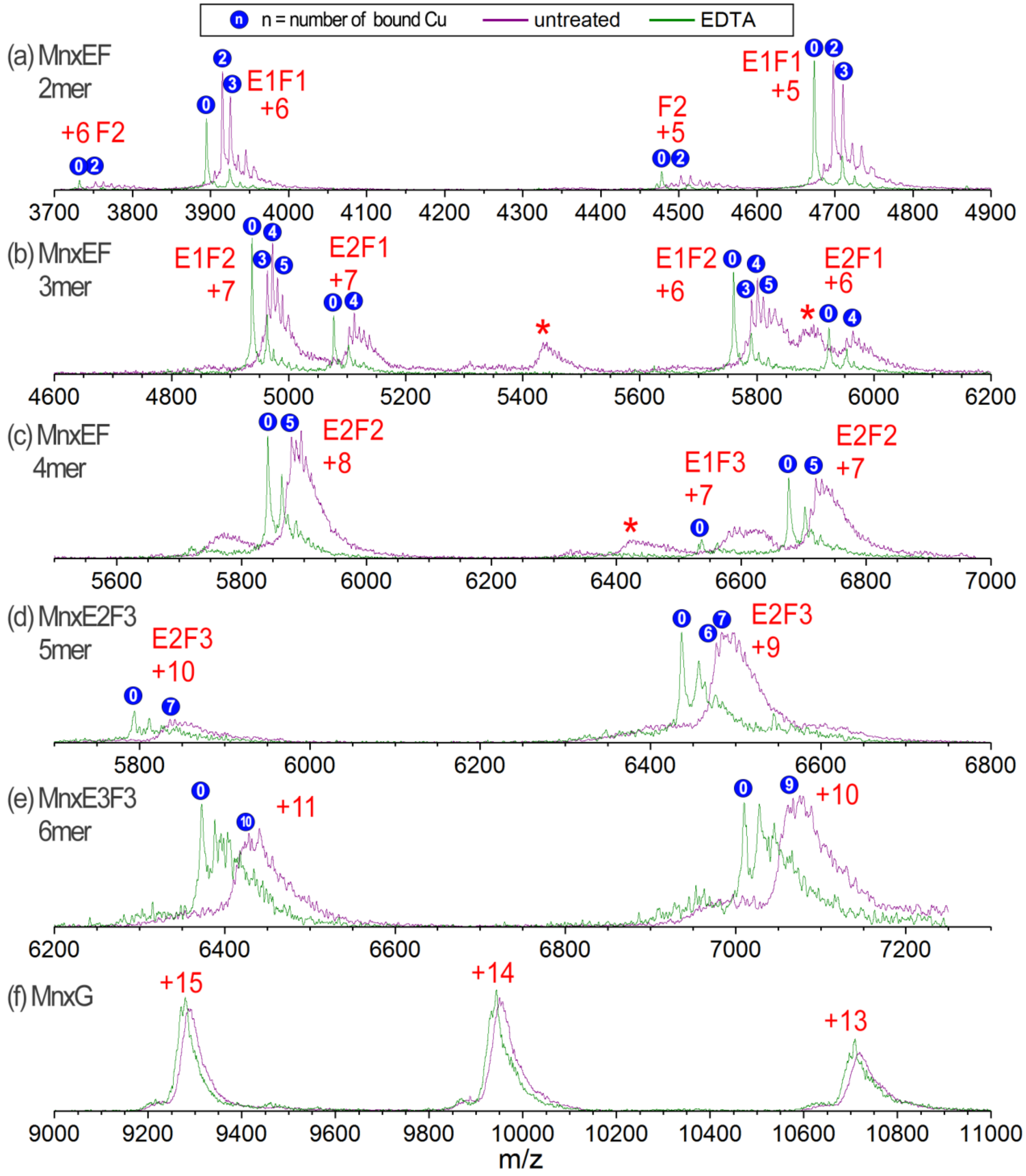

Supplementary Figure 4. Zoom-in mass spectra of Mnx components.

MnxE/F subcomplexes (a, b, c, d, e) and MnxG (f) released from Mnx by SID at a collision voltage of 100 V for untreated wild type Mnx (purple curve) and EDTA treated Mnx (olive curve). The major species detected are labeled along with the charge states. The peaks labeled with asterisks are partially unfolded MnxE3F3 hexamers that protruded into the region where the data were extracted from the 2D IM-MS spectra (the regions highlighted with red lines in Figure 3d). The major Cu binding species are labeled with blue circles, with estimated numbers of bound Cu annotated in the circles (for apo species the number is 0). Additional details are in Supplementary Note 3.

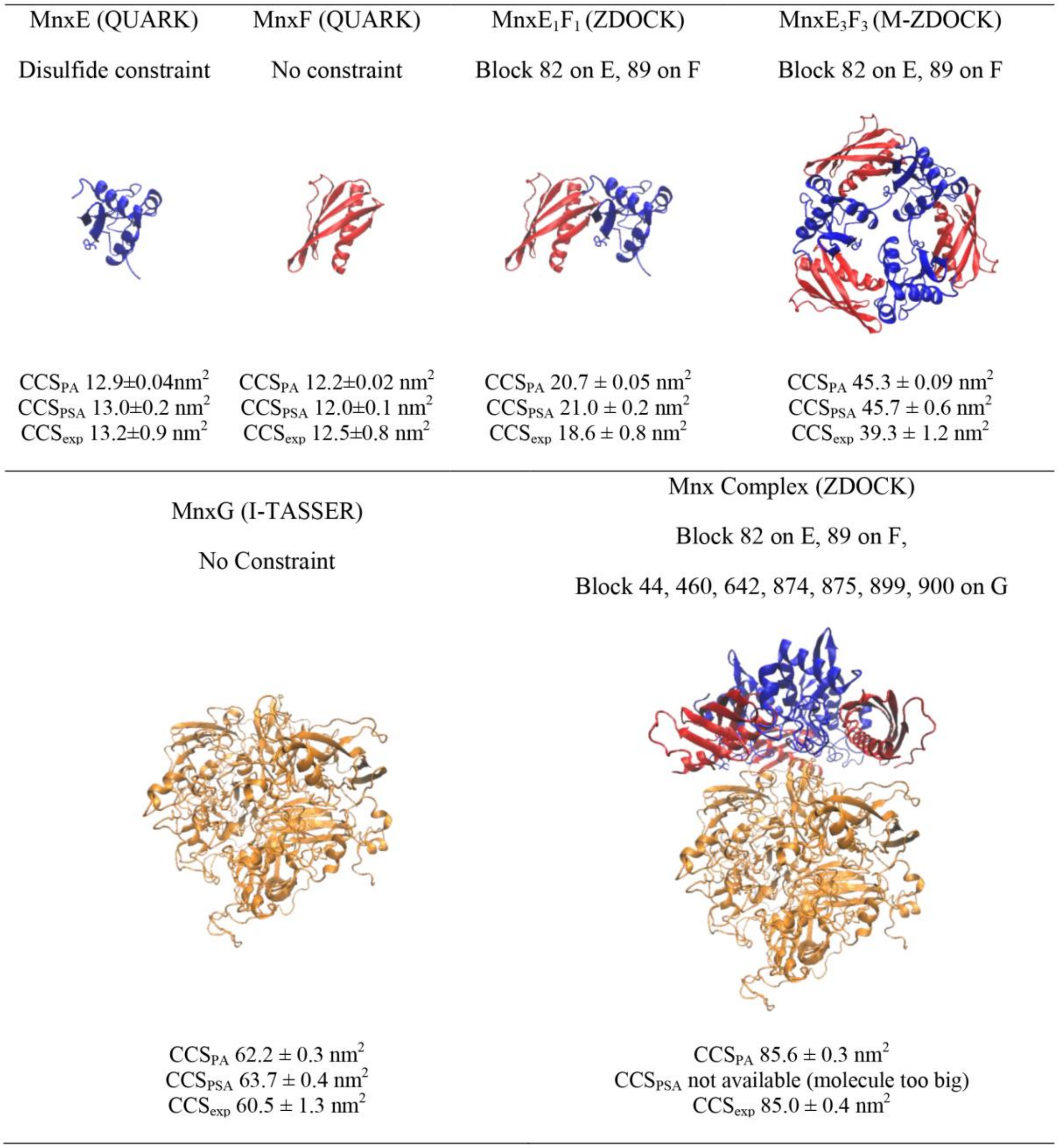

Supplementary Figure 5. Plausible models of Mnx and its substructures.

The *ab initio* models of MnxE and MnxF were docked into a MnxEF dimer and then a hexamer. The MnxEF hexamer model was docked onto the homology model of MnxG to generate a model for the complex. Specific residues (residue numbers listed above structures) were blocked from being at protein-protein interfaces in docking based on covalent labeling data. CCSs for most models calculated with two different algorithms (CCS_{PA} and CCS_{PSA}, details in supplementary method) are reasonably consistent with experimental values (CCS_{exp}), except for the MnxEF dimer and hexamer which may have collapsed significantly after being released from the complex. I-TASSER [1, 2] was used to generate the model of MnxG based on its sequence similarity to the characterized MCO, human ceruloplasmin.[3] The structures of MnxE and MnxF were modeled *de novo* and docked using online servers.[4, 5, 6]

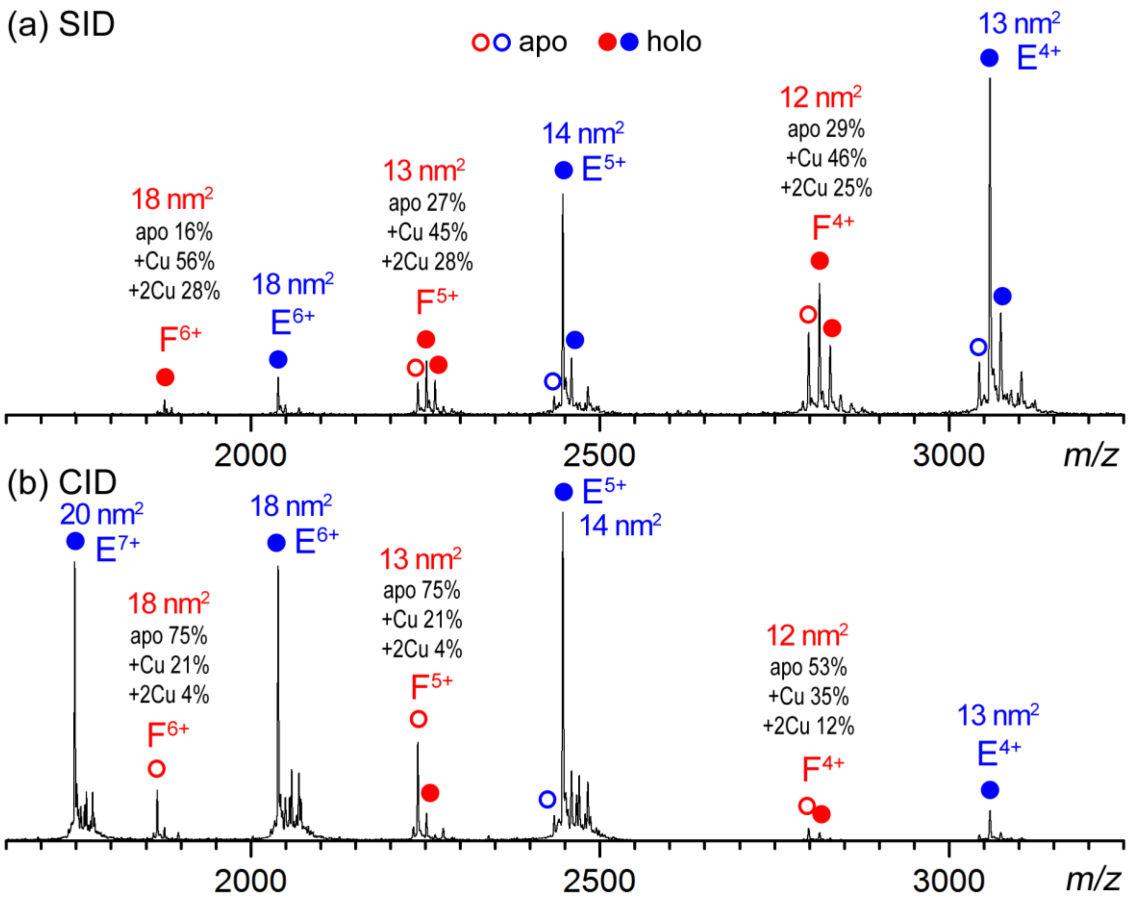

Supplementary Figure 6. Zoomed-in views of the mass spectra showing metal binding to MnxE and MnxF monomers.

These monomers were released from the intact Mnx complex in the gas phase by (a) SID and (b) CID. The CCSs (in nm^2) from ion mobility measurement are annotated next to the peaks (based on apex of drift time peak). While most of the released MnxE bound to one copper in both SID and CID, the copper load on MnxF differs significantly between SID and CID. The overall metal load on MnxE is 70~80% for one copper and 10~20% for two copper. For MnxF, about half of the protein is bound to one copper and ~25% is bound to two copper, regardless of charge state (4+ to 6+ in view). However, on average the MnxF released in CID showed less copper loading, with higher charge states (5+, 6+) binding less copper than the lower charge state (4+). The higher charged monomers also showed larger CCSs than the lower charged monomers. In Figure 3b, these same monomers created the spots that are enclosed by the parallelograms labeled with "extended" and "compact" near *m/z* 2000. Most of the monomers in CID have CCSs and drift times that place them in the "extended" parallelogram, implying extended and unfolded conformations. In contrast, most of the ions with *m/z* higher than 2000 in SID showed compact conformations (also in Figure 3d, parallelograms labeled with "MnxE/MnxF 1mer"). The MnxF 6+ ion in SID showed a lower copper load than the 5+ and 4+ ions in the spectrum above probably because it is partially unfolded. Even though the MnxF ions at 5+ and 4+ from CID showed compact CCS similar to those in SID at the same charge states, the lower copper load suggests they might have been unfolded during the dissociation process and then refolded after release from the complex. This observation is consistent with previous studies showing that significant subunit unfolding occurs during CID which may trigger metal loss. However, the energy-sudden SID activation minimizes unfolding and preserves the native ligands/metals bound to the subunits.

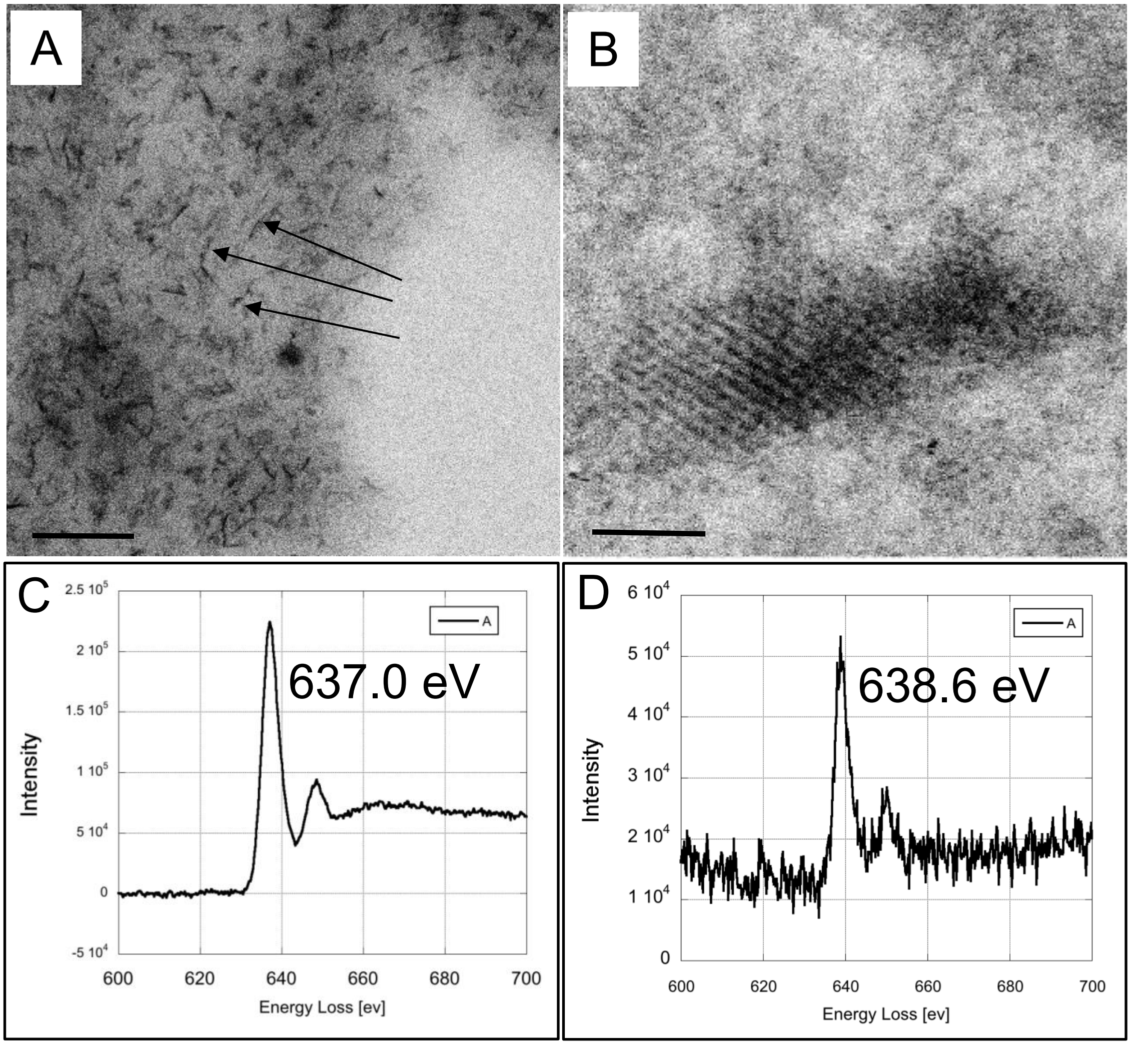

Supplementary Figure 7. EELS analysis of Mn particles' oxidation states.

(A) S/TEM image of newly formed Mn nanoparticles (arrows). (B) Representative Mn nanoparticle used for EELS analysis. (C) A spectrum of a Mn^{2+} standard ($MnCl_2$). (D) A representative spectrum from a single Mn nanoparticle, such as in (B). Ionization energy of $L_{2/3}$ edge increased by 1.6 eV compared to Mn^{2+} standard. Scale bars in (A, B) are 50 nm and 2 nm, respectively.

	CCS (nm^2)	CCS Relative Error	TEM Diameter (nm)
Mnx Complex	85.0	0.45%	7.9 ± 0.6
Released MnxE	13.2	6.57%	
Released MnxF	12.5	6.13%	
Released MnxG	60.5	2.11%	
Released MnxEF Hexamer	39.3	3.12%	6.8 ± 0.6
Released MnxEF Dimer	18.6	4.20%	

Supplementary Table 1. Size measurement of Mnx complex by IM-MS and by TEM.

Collisional cross section (CCS) was determined experimentally following a previously described protocol based on the drift time measured in the ion mobility mass spectrometry spectra [7]. Relative error was calculated from multiple measurements using different ion mobility parameters. TEM diameter was calculated based on the average diameter of particles in TEM images. The reported error is standard deviation. More than 1000 Mnx particles and 100 MnxEF hexamer particles were measured. Unlike the MS experiments, the MnxEF hexamer particles examined here were purified from a separate plasmid that did not contain any MnxG. A C-terminal strep tag was attached to MnxF for purification. The CCS from the IM-MS measurement for the Mnx complex was performed on the intact protein complex shown in Figure 1 without intentional activation. The CCSs for all other subunits and subcomplexes are measured on the released species in SID (Figure 3d).

	untreated		treated with EDTA		Theoretical Mass (apo) (Da)
	Experimental Mass (Da)	Delta Mass (Da)	Experimental Mas (Da)	Delta Mass (Da)	
Mnx	211216 ± 52	+2885	210268 ± 92	+1937	208331
Released MnxE 1mer	12169.7 ± 0.9<br>12231.3 ± 0.9<br>12292.3 ± 1.0	~ 0<br>+62.2 (1 Cu)<br>+123.2 (2 Cu)	12169.8 ± 0.8	~ 0	12169.1
Released MnxF 1mer	11193.0 ± 0.6<br>11254.4 ± 0.6<br>11315.8 ± 0.4	~ 0<br>+61.8 (1 Cu)<br>+123.2 (2 Cu)	11193.0 ± 0.7	~ 0	11192.6
Released MnxEF 2mer	22508 ± 1<br>23485 ± 1	+123 (2 Cu)<br>+123 (2 Cu)	22386 ± 2<br>23361 ± 2	~ 0<br>~ 0	F_2 22385<br>E_1F_1 23362
Released MnxEF 3mer	34799 ± 1<br>35777 ± 2	+245 (4 Cu)<br>+246 (4 Cu)	34555 ± 3<br>35532 ± 3	~ 0<br>~ 0	E_1F_2 34554<br>E_2F_1 35531
Released MnxEF 4mer	47029 ± 1	+306 (~ 5 Cu)	46724 ± 4	~ 0	E_2F_2 46723
Released MnxEF 5mer	58345 ± 4	+429 (~ 6-7 Cu)	57927 ± 4	+11	E_2F_3 57916
Released MnxEF 6mer	70751 ± 57	+666 (~ 10 Cu)	70103 ± 21	+18	E_3F_3 70085
Released MnxG 1mer	139324 ± 27	+1078	139190 ± 27	+944	138246

Supplementary Table 2. Experimental mass of the major subcomplexes released from wild-type Mnx complex during SID.

The details of the EDTA treatment are further described in Supplementary Note 3. The released subcomplexes from the EDTA treated Mnx implied that most of the copper from the MnxE and MnxF is removed (small amount of residual copper on MnxE, as shown in Supplementary Fig. 4). The experimental mass is calculated based on the peak apex of the smoothed mass spectra; the standard deviation is from the residual error in fitting the charge state distribution of the mass values at the peak apex and did not consider peak widths which significantly increase uncertainties in mass measurement for large ions. Most of the species from the Mnx with EDTA treatment showed good agreement with theoretical mass within experimental error, except for MnxG which has extra mass from the expected sequence and may have been modified. It is noted that many of the high mass ions have broad peak widths due to variable attachment of other species (copper, sodium, potassium, etc.) and variable stoichiometry of modified forms of MnxE/MnxF (*e.g.* a hexamer of E_3F_3 can have 1 of the E modified, or 2 of the E modified, *etc*.), which generates uncertainties in mass determination that are not represented by the error listed in the table. Some of the MnxEF multimeric species listed showed variable copper binding ratio; only the most abundant species are listed here. The copper binding stoichiometry of larger species cannot be confidently determined due to limited resolution and complication from nonspecific salt adducts (such as sodium ions) and protein modifications. Theoretical mass is calculated based on the confirmed sequence considering truncation for MnxE/MnxF based on top-down LC-MS data (Supplementary Fig. 1); the theoretical mass for MnxG is calculated based on the predicted sequence. Potential bound metal is not added to the theoretical mass. The Delta Mass column lists the mass difference between the experimental and theoretical values.

	MS	CID 120 V (Trap)	SID 120 V
SID Entrance 1 (V)	-110	-110	+10
SID Entrance 2 (V)	-125	-125	-95
SID Front Top (V)	-108	-108	-160
SID Front Bottom (V)	-111	-111	+20
SID Surface (V)	-113	-113	-95
SID Middle Bottom (V)	-115	-115	-125
SID Rear Top (V)	-111	-111	-270
SID Rear Bottom (V)	-110	-110	-125
SID Exit 1 (V)	-112	-112	-145
SID Exit 2 (V)	-135	-135	-115
Trap Bias (offset value)	45 (actual voltage ~ -105V)	45 (actual voltage ~ -105V)	175 (actual voltage ~ +25V)
CID CE (V, offset value)	4	120	4
Trap Gas (mL/min)	2	6	2

Capillary voltage 1.2 kV, Cone 20-50 V at room temperature
Helium cell gas 120 mL/min, IMS gas 60 mL/min, IMS traveling wave 20 V at 200 m/s
Backing pressure 2.4 mbar, Analyzer pressure 7×10^{-7} mbar

Supplementary Table 3. Experimental parameters for native MS and SID experiments on the modified Synapt G2s.

The collision voltage in SID is defined as the potential difference between the trap cell and the SID surface. The voltages on the SID device were controlled by an external power supply (Ardara Technologies, Ardara, PA, USA). The other voltages and gas flows were directly controlled by the instrument software.

Supplementary Note 1. Observed and predicted amino acid sequences and molecular weights (MWs) for Mnx proteins.

MnxD (predicted, not detected) | UniProt: A7KBU4 | NCBI: ABP68887 | MW: 29001 Da
MRHSDYLKNLLENRRNEVRQVDPIHEVGNHITSPPSATSLEENKIIAEYLEQKNLHTLAN
AVPEFKKPLILKKFFKMKRNQEVIVYIDHQDNTQEISGKVNAIGRDFVILTNLKDRIWIP
YKTILSANSPSGVPTYENAHQNFIYDNDLKRKLTTNFGETVANRDVLIQQFFEESLKGNL
HRWQGVWVKVFLADEIVIGKIASVTEEFILLQSFGSEREIALTDVTLIRSARLFIQFLLM
GKNMIKSMFR

MnxE full length (observed) | UniProt: A7KBU5 | NCBI: ABP68888 | MW: 12169 Da*
MHDSPLKSLSAASNVASVNDPLFDFFNKHMGKQILIITESSQLNILGQTFRPIFCGKVAE
VEPGHLTLSPVTIKILNAPFHKFPIPLSIPFEKIAHFTTDVDCSMRIPLV

MnxE 10-110 (observed) | UniProt: A7KBU5 | NCBI: ABP68888 | MW: 11160 Da*
SAASNVASVNDPLFDFFNKHMGKQILIITESSQLNILGQTFRPIFCGKVAE
VEPGHLTLSPVTIKILNAPFHKFPIPLSIPFEKIAHFTTDVDCSMRIPLV

MnxF (observed) | UniProt: A7KBU6 | NCBI: ABP68889 | MW: 11193 Da
STDYSKMTDVNEIHDSAILEHFRNGIGHKTLVISPSYPYMFVGIIKELIGDTV
MIDVETTHFAQLENREWYIHIHNIEVFYIERPGAPKIPKLEDY

MnxF (predicted) | UniProt: A7KBU6 | NCBI: ABP68889 | MW: 12013 Da
MEALFPMSTDYSKMTDVNEIHDSAILEHFRNGIGHKTLVISPSYPYMFVGIIKELIGDTV
MIDVETTHFAQLENREWYIHIHNIEVFYIERPGAPKIPKLEDY

MnxG (predicted) | UniProt: A7KBU7 | NCBI: ABP68890 | MW: 138246 Da
MLRKFHVVGISTRIVVNTFGDHNPNGRIYVLKENESKLKDLVRKNPYKPIDLVQPLAIRA
NEGDIVEILFENQLSFSAGMHFQEADYSVLSSDGADAGYNPDTTVEPGGEILYRLNVNQE
GICFFTDLGNVSSTEQGSSVQGLFGALLVQKRGSSWTDPVTGGPINSGVYADIHHPFLPS
FREYAWFFNDEMEIRDLTGERPLNPMTNQEAESFHGVNLRYEPMTNRKRLMEAGVVCPDC
DSEEVHHDSWVFGDPATPILRGYVGDPAVIRLIHGGVKETHVFHYHVHQWLGDSSNINAE
ILDAQSISPQTHYSIQPLYGLGSLHGAIGDSIIHCHLYPHFGIGMWGMNRVFDTLQDGSQ
CYPNGVRIKALMPLPDRPEPPKPTPEKPGFPNFIPGKVGYKAPRPPLGIVGGREMTELER
NAAIENPRPGAVFVDPCLDQDPVVVEFNVSAIEMPVVYNKQGWHDPKARFYVMDEDLDDI
LSGKKEPEPLVFHVPAGTCIRMNYTNRMPHILDGDAFQLVTRTYENGFHIHFVKFDVLAC
DGGNVGWNYDSAVLPGQTIRYEWYAETELKAFFFHDHLFANSHQQHGVFGAGVIQPRFSK
FLDSRTGDEVDHGTQISVEHPLIPDYRDQTLFVHDFALLFDKNGRPIQPPEYPGSEDDPG
VFGVNFKCEPLKFRLGEDCDPAYSFSSYVHGDPVTPILRAYEGDPIRIRLLQGAHEESHS
FNIHGLRWKEERPDLGSSMKAQQHIGISESFTFETEIPASGDYLWAFEDEEDVWLGTWGL
IRAYKGRMEDLIVLTDREALPEGSAETPKPTGKPPEKANPLASLPPGAYQGSPVKKFEVV
AFQTPIQYNSYGDHDPYGIIFALKEDVEDILTGKKNPVPLILRANVGDLVEVTLTSELKK
ELFPFQDGIHPYPPVKEQSFYPPSLRISLHTSLLNYDVKTSSGDTVGYNPDQTVGPGETI
TYRWFVDGQFGMCSMWDMADLRNHRSFGTFGAFVAESRFTTYLDPYSLEKAITGENVILR
HPLLPATREFVLILHDGVRLEDKDGKVIIDPMDGVVPDTEELEEVDTYDYGSRGFNYRSE
RLINRYKEHPVMHELFSSEVFGDPATPLFEAYPGEPVVMRITTPAERRRAHTFHLHGHYW
KFDSKDLDSRIQSFLGHMVTGHTDDLRLIGGAGGVFNFPGDYLYRSGNIRWDIELGMWGI
FRVHKDSKENLPRLEEVEGGWDNEEKA

The experimental mass of MnxF is 11.2 kDa which is truncated from the predicted sequence (truncated region highlighted in red). Top-down mass spectrometry showed that the MnxF started from the 2nd methionine in the sequence (Supplementary Fig. 1). The average MWs were obtained from a freeware Molecular Weight Calculator (https://omics.pnl.gov/software/molecular-weight-calculator) based on the sequences. The MW for MnxE (noted with asterisks) were subtracted by hydrogen losses (-2 Da) because of the detected mass shift assigned to disulfide bond formation by top-down MS.

Supplementary Note 2. Impurity in the Mnx sample (Supplemental details for Figure 1)

The impurity species in Figure 1b was isolated and activated by SID in a manner similar to that described for Mnx in the main text. The dissociation pattern suggested that it was a symmetric 166 kDa hexamer consisting of monomers at 27 kDa [8]. Bottom-up LC-MS analysis of the tryptic peptides identified the presence of a putative GTP cyclohydrolase 1 type 2 from *E. coli* (UniProt ID: P0AFP7, data not shown). The known structure of this protein is a hexamer with the monomer mass of 26.9 kDa. Therefore, we believe the impurity species is the putative GTP cyclohydrolase from the host cell that co-purified with wild-type Mnx following the heat denaturation protocol.[9] When using affinity purification protocol for the construct where the C-terminus of MnxG is strep-tagged, we did not observe this impurity protein in the final sample with mass spectrometry.

Supplementary Note 3. Additional details of EDTA treated Mnx for Supplementary Figure 4.

The EDTA treatment was performed by incubating ~3 mg/mL Mnx complex in 100 mM NH_4OAc buffer for 2-3 days at 4°C and then incubating it in 2mM EDTA ammonium salt (balanced to pH 7) for ~10 minutes at ambient temperature. The buffer was then exchanged to 100 mM NH_4OAc to remove EDTA. The spectra were acquired with a protein concentration of ~1 mg/mL. After EDTA treatment, the majority of the bound Cu on MnxE and MnxF was removed, as manifested by the downward mass shift of the EDTA spectra relative to the untreated spectra. In untreated samples (Supplementary Fig. 4a – e), the combination of bound Cu and protein modifications give rise to complex spectra with significant broadened peaks which can hardly be resolved for MnxEF multimers larger than the 3mer. The numbers of bound Cu shown are estimates based on mass shifts. Removing the bound Cu with EDTA greatly simplified the spectra, allowing the subunit stoichiometry of MnxE/F in the subcomplexes to be confidently determined. The most abundant species in the EDTA treated samples exclusively match the theoretical mass of apo MnxEF multimers. Additional peaks at higher mass correspond to MnxEF multimers containing modified MnxE and/or MnxF monomers, and to species carrying nonspecifically bound salt/solvent. For MnxG (Supplementary Fig. 4f), the relatively small mass shift after EDTA treatment did not allow confident determination of the change in the number of bound Cu (peak apex shifted by ~130 Da).

Supplementary Note 4. Structural characterization of MnxEF (Supplemental details for main manuscript)

Native mass spectrometry was attempted on solutions of MnxEF hexamer that were overexpressed and purified in the absence of MnxG. However, the experiment was not reproducible because MnxEF is highly unstable, especially in the ammonium acetate buffer that is optimal for mass spectrometry. Instead, MnxEF was separated from the Mnx complex *in situ* after SID and ion mobility so that information on the copper binding affinities of MnxE and MnxF could be obtained. MnxF exhibits more variable Cu loading than MnxE. MnxF binds 1-3 Cu atoms per subunit, on average more than one, while MnxE appears to bind only one Cu per subunit. The binding of Cu on MnxE was even maintained in CID where the MnxE partially unfolded (Supplementary Fig. 6). We hypothesize that this variability results from the location of these metals' binding sites within the secondary and/or tertiary structure of their subunits. The major metal binding site in MnxE is likely localized at several residues at the N-terminus which may not require a specific tertiary fold, therefore the metal binding is not directly affected by unfolding.[10] In contrast, metal binding on MnxF is affected by protein unfolding, with more highly

charged and more extended conformations retaining less copper (Supplementary Fig. 6). If this hypothesis is true, then the compact MnxE/MnxF monomers released in SID can better resemble the native structure and metal binding properties in the Mnx complex than the unfolded monomers.

Previous reports found that the copper content of Mnx varies [11, 12, 13]. The ability to extract metals from the protein with chelators (such as EDTA, Supplementary Fig. 4, Supplementary Table 2) explains why Mnx has been shown to have a varying copper load. ICP-OES measured ~10 Cu / Mnx in Tris Buffer, and ~15 Cu / Mnx in HEPES. These discrepancies were attributed to sample preparation methods (type of buffer, presence of chelating reagent), although we propose that the labile nature of Cu bound to MnxF may also cause these variations. The bound Cu on MnxF can be more easily removed by EDTA than the Cu atom(s) on MnxE, and significant loss of Cu on MnxF can be observed when Mnx is stored in Cu-free buffer for extended period of time. Nonetheless, for Mnx prepared in HEPES, we estimate ~10 Cu bound to MnxEF hexamer based on SID results and it is well-established that MCOs (like MnxG) contain at least 4 Cu[14]. The stoichiometry is qualitatively consistent with our previous ICP result. Yet further improvements in high resolution native MS are required to better define the Cu binding stoichiometry in MnxG.

Supplementary Methods

Top-down MS (Experimental details for Supplementary Fig. 1)

The purified protein sample was denatured and analyzed with top-down LC-MS. The Waters NanoAcquity liquid chromatography was equipped with C5 reversed phase column (5 µm, 300 Å, inner diameter 100 µm, length ~ 50 cm, solvent A: 2.5% isopropanol, 5% acetonitrile, 0.58% acetic acid, 0.01% trifluoroacetic acid: solvent B: 45% isopropanol, 45% acetonitrile, 0.58% acetic acid, 0.01% trifluoroacetic acid), and was coupled to a Thermo Orbitrap Elite mass spectrometer for standard data-dependent acquisition of the intact proteins with higher-energy collisional dissociation (HCD) fragmentation at a normalized collision energy of 25 V. The major species detected corresponded to the MnxE and MnxF subunits (Supplementary Fig. 1a, b, c). The MnxG subunit was not detected, but a peak in the chromatogram around 41 min showed high baseline with unresolved species in MS (Supplementary Fig. 1d). It is speculated that this early eluting species, not resolved in MS, originated from MnxG which was not fully denatured. Therefore, it was not retained strongly on the column and did not ionize well within the detection range of the mass spectrometer. It is noted that even when the protein was denatured in 50% organic solvent for direct infusion into a mass spectrometer, the MnxG subunit could not be detected (data not shown). The MnxG subunit can be detected in standard bottom-up experiment from its tryptic peptides and can also be detected when it is released from the complex by SID in native MS. The cause of this unusual behavior remains to be explored.

The data were analyzed with MSAlign[15] for protein identification. The protein database included reviewed *E. coli* K-12 proteins in UniProt, appended with the MnxD, MnxE, MnxF, and MnxG sequences. MnxD was not detected in top down or native MS, consistent with a previous report [9] that MnxD is not part of the active complex even though the gene was encoded into the construct for protein expression. The coverage map of MnxE is listed in Supplementary Fig. 1b and showed limited fragmentation on the latter half of the sequence with a modification of –2 Da. This implies that there is a disulfide linkage between the two cysteines in the protein which protected the protein from fragmentation, leading to limited coverage in the region with the cysteine resides and a mass shift of –2 Da from the expected sequence. For MnxF (Supplementary Fig. 1c), an N-terminal truncation of the first 7 residues was detected consistently across all the samples prepared from different batches. The experimentally confirmed MnxE and MnxF sequences including the modifications were used for interpreting all the other experimental data.

Covalent labeling and Computational modeling (Experimental details for Supplementary Fig. 5)

Mnx complex was reacted with acetic anhydride or N-hydroxysuccinimidobiotin (NHS-biotin) to label surface exposed lysine residues. For labeling with acetic anhydride, a final concentration of 10 mM labeling reagent was added to 10 µL protein (10 µM) and reacted for 20 min. For labeling with NHS-biotin, the final concentration of 1.5 mM EZ-Link NHS-biotin (Thermo) was reacted with 6.25 µM Mnx complex for 30 min at room temp. The labeled protein was digested with pepsin and the digests were analyzed by LC-MS on a dual linear ion trap Velos Pro mass spectrometer (Thermo Fisher Scientific) coupled to a nanoACQUITY UPLC (Waters). Identifications were made by SEQUEST HT in Proteome Discoverer software (v1.4). The database searched included the sequences of *E.coli* host proteins, Mnx and keratin. No enzymes were specified for *in silico* digestion. Dynamic modifications were lysine acetylation (K+ 42.01057) or lysine biotinylation (K+ 226.07760). Peculator was used to validate the

identities of peptides with a 1% false discovery rate (FDR). Additionally, the identity of the labeled peptides was manually confirmed.

The overlapping set of labeled residues that were found with both labeling reagents was used in the modeling experiment as constraints. The labeled residues used are: 82 for MnxE; 89 for MnxF; 44, 460, 642, 874, 875, 899, 900 for MnxG. The MnxG model was generated with I-TASSER [1, 2] based on homology to existing structures in PDB, chiefly that of human ceruloplasmin [16], the characterized MCO with the strongest sequence similarity to MnxG. The top model in the output was used for further studies. The MnxE and MnxF models were generated with the *ab initio* prediction tool QUARK, with the experimentally confirmed protein sequence. The two cysteines in MnxE were set to be less than 7 Å apart to account for the disulfide bond. No constraint was put on MnxF. Most of the generated MnxE and MnxF monomer models are consistent with experimental collisional cross sections (CCSs) from ion mobility experiments (Supplementary Table 1) within experimental error, therefore it is difficult to filter the models solely based on CCS. The top MnxE and MnxF models were chosen and docked into MnxEF dimer with K82 on MnxE and K89 on MnxF blocked from contact. The top MnxEF dimer model was docked into MnxEF hexamer with 3-fold symmetry with M-ZDOCK and with the same residues blocked. Finally, the top five MnxEF hexamer models were docked onto the MnxG I-TASSER model with the aforementioned MnxG residues blocked. This simulation generated the models for the Mnx complex. Some of the structures had very small contact and large exposed surfaces and were therefore discarded. The theoretical CCS of the top scored model was in good agreement with the experimental CCS and is shown in Supplementary Fig. 5 along with the models of the subcomplexes and the monomeric subunits in Mnx. The theoretical CCS values for the models were calculated using IMPACT [17] with the Projection Approximation (PA), scaled by an empirical factor of 1.14 to fit experimental data [18]. The theoretical CCS values were also calculated by Projected Superposition Approximation (PSA), which included a more sophisticated algorithm than PA to better capture the molecular shape while maintaining the calculation at a reasonable speed for large molecules[19]. For the MnxEF dimer and hexamer in particular, the CCS values of the models are significantly larger than the experimental values. The discrepancy could be explained by the collapse of the released subcomplexes which are still folded but may have rearranged after losing their binding partners. Similar behavior has been observed for other large subcomplexes released in SID[20]. Therefore, the CCS values of these subcomplexes could not be used as experimental constraints for selecting the models.

It is important to note that this is only one of the plausible models that fit the limited number of experimental constraints. Some of the models other than the top hit from the output can also match the experimental CCS with reasonable subunit connectivity and similar overall topology to the structure shown here, but with different structural details and contacts. In particular, the lack of known structures for MnxE and MnxF monomers poses significant challenges for building high-confidence models of the Mnx complex, because differences in the structures of the fundamental units can lead to pronounced differences in the structure of the final protein complex. However, even with the limited information, it can be concluded that the structural model proposed, where MnxE and MnxF form a symmetric hexamer attached to MnxG, is the most reasonable structure given the subunit connectivity from SID results and the experimental size of the Mnx complex. A recent computational study for protein structure proposed two major topologies for protein complexes with two unique subunits and C3 symmetry: a ring structure consisting of three dimers of the two unique subunits, or a triangle structure with a homo-trimer of the unique subunit in the center [21]. The lack of strong $MnxE_3$ or $MnxF_3$ trimers in the released subcomplexes favors the topology of a ring structure with alternating repeats of MnxE and MnxF, which is in agreement with the highly scored models from the docking simulations. To generate models at higher confidence in the absence of high resolution data from conventional techniques, it is essential to obtain

more constraints from experiments. We will pursue this information through additional labeling experiments, crosslinking, and computational efforts exploring larger conformational space. Our initial crosslinking experiments with bis(sulfosuccinimidyl) 2,2,4,4-glutarate (BS2G) and bis(sulfosuccinimidyl)suberate (BS3) did not generate confident identifications for inter-subunit crosslinks due to low efficiency of crosslinking. Crosslinking experiments with paraformaldehyde and glutaraldehyde were also not able to effectively capture multimers of MnxE and MnxF, and using high concentrations of these reagents resulted in excessive crosslinking and caused precipitation.

Supplementary References

1. Roy A, Kucukural A, Zhang Y. I-TASSER: a unified platform for automated protein structure and function prediction. *Nat Protoc* **5**, 725-738 (2010).

2. Zhang Y. I-TASSER server for protein 3D structure prediction. *BMC Bioinformatics* **9**, 40 (2008).

3. Brown MA, Stenberg LM, Mauk AG. Identification of catalytically important amino acids in human ceruloplasmin by site-directed mutagenesis. *FEBS Lett* **520**, 8-12 (2002).

4. Pierce BG, Wiehe K, Hwang H, Kim BH, Vreven T, Weng Z. ZDOCK server: interactive docking prediction of protein-protein complexes and symmetric multimers. *Bioinformatics* **30**, 1771-1773 (2014).

5. Xu D, Zhang Y. Ab initio protein structure assembly using continuous structure fragments and optimized knowledge-based force field. *Proteins* **80**, 1715-1735 (2012).

6. Yang J, Yan R, Roy A, Xu D, Poisson J, Zhang Y. The I-TASSER Suite: protein structure and function prediction. *Nat Methods* **12**, 7-8 (2015).

7. Bush MF, Hall Z, Giles K, Hoyes J, Robinson CV, Ruotolo BT. Collision Cross Sections of Proteins and Their Complexes: A Calibration Framework and Database for Gas-Phase Structural Biology. *Anal Chem* **82**, 9557-9565 (2010).

8. Song Y. Protein Primary and Quaternary Structure Elucidation by Mass Spectrometry. PhD Thesis. Ohio State University (2015).

9. Butterfield CN, Soldatova AV, Lee S-W, Spiro TG, Tebo BM. Mn(II,III) oxidation and MnO2 mineralization by an expressed bacterial multicopper oxidase. *Proceedings of the National Academy of Sciences* **110**, 11731-11735 (2013).

10. Zhou M, *et al*. Unveiling the Heterogeneity of an Uncharacterized Manganese Oxidizing Multicopper Oxidase Using High Resolution Native Mass Spectrometry and Surface Induced Dissociation. In: *The American Society for Mass Spectrometry 64th Conference*, 279055 (2016).

11. Butterfield CN, Tebo BM. Substrate specificity and copper loading of the manganese-oxidizing multicopper oxidase Mnx from Bacillus sp. PL-12. *Metallomics* **9**, 183- 191. (2017).

12. Butterfield C. Characterizing the Mn(II) oxidizing enzyme from the marine bacillus sp. PL-12 spore. PhD Thesis. Oregon Health & Science University (2014).

13. Tao L, *et al.* Copper Binding Sites in the Manganese-Oxidizing Mnx Protein Complex Investigated by Electron Paramagnetic Resonance Spectroscopy. *J Am Chem Soc*, (2017). **DOI:** 10.1021/jacs.7b02277

14. Solomon EI, Sundaram UM, Machonkin TE. Multicopper Oxidases and Oxygenases. *Chem Rev* **96**, 2563-2606 (1996).

15. Liu X, *et al.* Protein Identification Using Top-Down Spectra. *Molecular & Cellular Proteomics* **11**, (2012).

16. Bento I, Peixoto C, Zaitsev VN, Lindley PF. Ceruloplasmin revisited: structural and functional roles of various metal cation-binding sites. *Acta Crystallogr D Biol Crystallogr* **63**, 240-248 (2007).

17. Marklund EG, Degiacomi MT, Robinson CV, Baldwin AJ, Benesch JL. Collision cross sections for structural proteomics. *Structure* **23**, 791-799 (2015).

18. Benesch JL, Ruotolo BT. Mass spectrometry: come of age for structural and dynamical biology. *Curr Opin Struct Biol* **21**, 641-649 (2011).

19. Bleiholder C, Wyttenbach T, Bowers MT. A novel projection approximation algorithm for the fast and accurate computation of molecular collision cross sections (I). Method. *International Journal of Mass Spectrometry* **308**, 1-10 (2011).

20. Mashalidis EH, *et al.* High-resolution identification of human adiponectin oligomers and regulation by pioglitazone in type 2 diabetic patients. *Anal Biochem* **437**, 150-160 (2013).

21. Ahnert SE, Marsh JA, Hernandez H, Robinson CV, Teichmann SA. Principles of assembly reveal a periodic table of protein complexes. *Science* **350**, aaa2245 (2015).
